# Current Tobacco Smoking and Desire to Quit Smoking Among Students Aged 13–15 Years — Global Youth Tobacco Survey, 61 Countries, 2012–2015

**DOI:** 10.15585/mmwr.mm6620a3

**Published:** 2017-05-26

**Authors:** René A. Arrazola, Indu B. Ahluwalia, Eugene Pun, Isabel Garcia de Quevedo, Stephen Babb, Brian S. Armour

**Affiliations:** ^1^Office on Smoking and Health, National Center for Chronic Disease Prevention and Health Promotion, CDC; ^2^CDC Foundation, Atlanta, GA.

Tobacco use is the world’s leading cause of preventable morbidity and mortality, resulting in nearly 6 million deaths each year (*1*). Smoked tobacco products, such as cigarettes and cigars, are the most common form of tobacco consumed worldwide (*2*), and most tobacco smokers begin smoking during adolescence (*3*). The health benefits of quitting are greater for persons who stop smoking at earlier ages; however, quitting smoking at any age has health benefits (*4*). CDC used the Global Youth Tobacco Survey (GYTS) data from 61 countries across the six World Health Organization (WHO) regions from 2012 to 2015 to examine the prevalence of current tobacco smoking and desire to quit smoking among students aged 13–15 years. Across all 61 countries, the median current tobacco smoking prevalence among students aged 13–15 years was 10.7% (range = 1.7%, Sri Lanka to 35.0%, Timor-Leste). By sex, the median current tobacco smoking prevalence was 14.6% among males (range = 2.9%, Tajikistan to 61.4%, Timor-Leste) and 7.5% among females (range = 1.6%, Tajikistan to 29.0%, Bulgaria). In the majority of countries assessed, the proportion of current tobacco smokers who desired to quit smoking exceeded 50%. These findings could be used by country level tobacco control programs to inform strategies to prevent and reduce youth tobacco use (*1*,*4*).

GYTS is a nationally representative school-based, paper and pencil, cross-sectional survey of students in school grades associated with ages 13–15 years. GYTS uses a standardized methodology that allows for cross-country comparisons.* For this report, countries were selected if they met the following criteria: 1) nationally representative data (rather than subnational data) were available to allow for cross-country comparisons; and 2) data were collected during 2012–2015 to allow for estimation of recent prevalence estimates. Based on these criteria, 61 countries from all six WHO regions were selected for analyses.^†^ The number of participating countries from each WHO region were African Region (AFR, 10 countries)^§^; Eastern Mediterranean Region (EMR, 10)^¶^; European Region (EUR, 18)**; Region of the Americas (AMR, 13)^††^; South East Asian Region (SEAR, 5)^§§^; and Western Pacific Region (WPR, 5).^¶¶^ Overall sample sizes ranged from 534 students in San Marino to 10,018 in Bosnia and Herzegovina (median = 2,428), and overall response rates ranged from 60.3% in Nicaragua to 99.2% in Sudan. Data were weighted for each country to yield nationally representatives estimates of youths attending school.

Students were asked about current (past 30-day) use of cigarettes*** and any form of smoked tobacco other than cigarettes.^†††^ Current tobacco smoking was defined as smoking cigarettes or other smoked tobacco products on ≥1 day during the past 30 days. Students were classified as having a desire to quit smoking^§§§^ if they answered “yes” to the question, “Do you want to stop smoking now?”

Overall country-specific prevalence estimates with corresponding 95% confidence intervals were calculated for current tobacco smoking and desire to quit smoking. Estimates based on unweighted sample sizes <35 or relative standard error >0.3 are not reported. For countries where data are reported for both sexes, chi-squared tests were used to determine statistically significant differences (p<0.05) in current tobacco smoking between males and females.

Across all countries, the median current tobacco smoking prevalence among students aged 13–15 years was 10.7% (range = 1.7%, Sri Lanka to 35.0%, Timor-Leste). By WHO region, current tobacco smoking prevalence in AFR ranged from: 6.1% (Mozambique) to 20.2% (Seychelles); in EMR, from 7.2% (Pakistan) to 23.3% (Jordan); in EUR, from 2.4% (Tajikistan) to 27.4% (Bulgaria); in AMR, from 5.8% (Paraguay) to 22.0% (Argentina); in SEAR, from 1.7% (Sri Lanka) to 35.0% (Timor-Leste); and in WPR, from 3.5% (Vietnam) to 14.5% (Philippines) (Table).

**TABLE:** Prevalence of current tobacco smoking,* overall and by sex, among students aged 13–15 years — 61 countries, Global Youth Tobacco Survey, 2012–2015.

World Health Organization region/country	Survey year	Overall unweighted sample size	Prevalence of current tobacco smoking		
			Overall % (95% CI)	Males % (95% CI)	Females % (95% CI)
**African Region**					
Algeria	2013	4,023	7.4 (6.3–8.7)	14.9 (12.3–17.9)	1.8 (1.3–2.7)^†^
Cameroon	2014	1,873	7.4 (4.8–11.5)	10.3 (6.8–15.4)	4.0 (2.4–6.6)^†^
Comoros	2015	1,551	9.1 (6.3–13.0)	13.2 (8.8–19.4)	5.6 (3.3–9.4)^†^
Gabon	2014	788	7.6 (6.1–9.5)	7.9 (6.3–9.8)	7.0 (5.1–9.5)
Kenya	2013	1,326	7.0 (4.9–9.8)	9.6 (6.6–13.8)	4.0 (2.2–7.2)^†^
Mozambique	2013	3,062	6.1 (4.7–7.9)	5.5 (4.0–7.5)	6.2 (4.4–8.7)
Senegal	2013	796	7.8 (5.0–12.1)	9.7 (5.9–15.7)	—^§^
Seychelles	2015	1,525	20.2 (17.2–23.7)	25.6 (21.7–30.0)	15.2 (11.9–19.2)^†^
Togo	2013	2,801	6.9 (5.3–8.9)	9.8 (7.3–13.0)	2.7 (1.8–4.2)^†^
Zimbabwe	2014	5,114	16.2 (10.6–24.1)	17.3 (11.4–25.5)	12.8 (7.9–19.9)^†^
**Eastern Mediterranean Region**					
Bahrain	2015	2,465	15.7 (11.1–21.8)	22.7 (17.4–28.9)	8.5 (6.5–11.0)^†^
Djibouti	2013	1,361	11.6 (8.8–15.2)	13.0 (9.1–18.1)	9.1 (5.9–13.6)
Egypt	2014	2,141	10.1 (6.7–15.0)	16.3 (10.0–25.6)	—
Iraq	2014	1,266	11.1 (7.2–16.8)	16.2 (10.3–24.7)	6.0 (4.2–8.4)^†^
Jordan	2014	1,899	23.3 (17.7–29.9)	32.8 (27.6–38.4)	13.4 (9.1–19.4)^†^
Pakistan	2013	5,832	7.2 (5.8–9.0)	9.2 (7.1–11.7)	4.1 (2.8–5.9)^†^
Qatar	2013	1,716	12.3 (8.8–17.0)	18.4 (14.1–23.7)	6.2 (4.4–8.8)^†^
Sudan	2014	1,450	8.3 (6.3–11.0)	10.6 (7.7–14.4)	5.0 (3.0–8.2)^†^
United Arab Emirates	2013	3,376	10.5 (7.9–13.9)	14.6 (10.7–19.5)	6.4 (4.3–9.5)^†^
Yemen	2014	1,634	15.1 (10.9–20.5)	19.4 (14.5–25.5)	7.9 (4.5–13.7)^†^
**European Region**					
Albania	2015	3,482	9.4 (7.9–11.1)	12.9 (10.7–15.6)	5.6 (4.2–7.5)^†^
Belarus	2015	2,428	9.4 (7.5–11.7)	8.9 (6.1–12.8)	9.9 (7.8–12.6)
Bosnia and Herzegovina	2013	10,018	15.1 (12.9–17.7)	17.8 (15.2–20.7)	12.2 (9.7–15.3)^†^
Bulgaria	2015	3,532	27.4 (22.8–32.5)	25.7 (19.5–33.1)	29.0 (24.7–33.8)
Georgia	2014	962	10.0 (7.0–14.1)	13.9 (9.9–19.2)	—
Greece	2013	4,096	13.3 (11.4–15.4)	14.9 (12.9–17.1)	11.6 (9.5–14.1)^†^
Italy	2014	1,428	23.4 (20.8–26.4)	20.6 (16.6–25.3)	26.3 (22.3–30.1)
Kazakhstan	2014	1,715	2.8 (2.0–3.9)	3.5 (2.2–5.3)	1.9 (1.2–3.2)
Kyrgyzstan	2014	3,468	3.7 (2.7–5.0)	5.5 (3.9–7.9)	2.0 (1.2–3.1)^†^
Latvia	2014	4,025	23.3 (21.6–25.0)	23.7 (21.6–26.0)	22.7 (20.4–25.1)
Lithuania	2014	3,113	26.4 (22.9–30.1)	28.6 (24.5–33.2)	24.1 (20.6–27.9)^†^
Moldova	2013	3,548	8.3 (6.3–10.9)	12.7 (9.3–17.0)	3.8 (2.6–5.7)^†^
Montenegro	2014	3,692	8.4 (4.7–14.7)	—	4.2 (2.7–6.4)
Portugal	2013	7,600	13.9 (12.5–15.4)	12.8 (11.3–14.5)	15.1 (13.2–17.1)^†^
Romania	2013	3,328	11.2 (9.3–13.4)	12.2 (9.9–14.8)	10.1 (7.9–12.8)
San Marino	2014	534	14.6 (11.2–19.0)	14.4 (10.1–20.0)	15.0 (10.2–21.4)
Serbia	2013	3,076	15.0 (12.4–18.0)	15.3 (12.9–18.0)	14.6 (11.1–18.9)
Tajikistan	2014	2,411	2.4 (1.7–3.5)	2.9 (1.9–4.5)	1.6 (1.0–2.6)
**Region of the Americas**					
Argentina	2012	2,069	22.0 (18.5–26.0)	20.2 (17.6–23.0)	23.7 (18.5–29.7)
Bahamas	2013	1,033	10.7 (7.4–15.4)	13.8 (8.4–21.8)	6.9 (4.4–10.7)^†^
Barbados	2013	1,306	12.6 (10.4–15.3)	15.7 (12.2–19.9)	9.3 (7.1–12.0)^†^
Belize	2014	1,273	11.5 (9.5–13.9)	15.7 (12.2–20.0)	7.5 (5.4–10.4)^†^
Costa Rica	2013	2,158	8.3 (6.6–10.4)	9.0 (6.9–11.6)	7.6 (5.6–10.3)
El Salvador	2015	2,567	12.2 (10.0–14.7)	14.7 (11.7–18.3)	9.4 (7.3–12.1)^†^
Guatemala	2015	3,351	15.7 (13.6–18.2)	18.0 (15.1–21.4)	13.2 (10.6–16.3)^†^
Guyana	2015	1,000	11.7 (8.6–15.7)	16.1 (10.8–23.2)	7.5 (4.5–12.5)^†^
Nicaragua	2014	3,006	14.6 (12.8–16.7)	16.8 (14.0–20.0)	12.3 (10.2–14.8)^†^
Panama	2012	4,077	8.1 (7.3–9.1)	10.3 (9.1–11.6)	6.2 (5.1–7.4)^†^
Paraguay	2014	5,153	5.8 (4.8–6.9)	5.9 (4.7–7.4)	5.7 (4.5–7.1)
Peru	2014	2,299	9.0 (6.4–12.5)	10.5 (7.2–15.2)	7.4 (5.2–10.5)^†^
Uruguay	2014	3,256	9.9 (8.3–11.8)	9.6 (7.6–12.1)	9.8 (8.0–11.9)
**South East Asian Region**					
Bhutan	2013	1,378	16.6 (13.9–19.4)	26.3 (21.6–31.6)	8.6 (7.0–10.6)^†^
Indonesia	2014	4,317	19.4 (15.0–24.8)	35.3 (27.4–44.0)	3.4 (2.2–5.3)^†^
Sri Lanka	2015	1,416	1.7 (0.9–3.2)	—	—
Thailand	2015	1,721	14.0 (10.4–18.6)	20.7 (16.0–26.3)	7.1 (4.4–11.2)^†^
Timor-Leste	2013	1,908	35.0 (28.9–41.6)	61.4 (48.1–73.2)	15.4 (12.0–19.5)^†^
**Western Pacific Region**					
Brunei	2013	917	10.2 (6.3–16.0)	15.0 (8.5–25.1)	5.1 (2.7–9.7)^†^
Mongolia	2014	6,178	5.6 (4.7–6.7)	8.2 (6.7–9.9)	3.0 (2.1–4.1)^†^
Philippines	2015	5,885	14.5 (11.6–18.0)	20.5 (16.3–25.4)	9.1 (6.2–13.3)^†^
South Korea	2013	3,437	5.9 (4.7–7.3)	8.4 (6.6–10.7)	3.1 (2.1–4.4)^†^
Vietnam	2014	3,430	3.5 (2.6–4.7)	6.3 (4.6–8.4)	—

**Abbreviation:** CI = confidence interval.

* Current tobacco smoking was defined as answering ≥1 day to the question “During the past 30 days, on how many days did you smoke cigarettes?” and/or “Yes” to “During the past 30 days, did you use any form of smoked tobacco products other than cigarettes (such as [country fills appropriate examples])?”

^†^ Female prevalence significantly different from males at p<0.05.

^§^ Data not reported because unweighted sample size <35 or relative standard error >0.3.

By sex, the median current tobacco smoking prevalence was 14.6% among males (range = 2.9%, Tajikistan to 61.4%, Timor-Leste) and 7.5% among females (range = 1.6%, Tajikistan to 29.0%, Bulgaria). Among males, the prevalence of current tobacco smoking by WHO region ranged from 5.5% (Mozambique) to 25.6% (Seychelles) in AFR; 9.2% (Pakistan) to 32.8% (Jordan) in EMR; 2.9% (Tajikistan) to 28.6% (Lithuania) in EUR; 5.9% (Paraguay) to 20.2% (Argentina) in AMR; 20.7% (Thailand) to 61.4% (Timor-Leste) in SEAR; and 6.3% (Vietnam) to 20.5% (Philippines) in WPR (Table). Among females, the prevalence of current tobacco smoking by WHO region ranged from 1.8% (Algeria) to 15.2% (Seychelles) in AFR; 4.1% (Pakistan) to 13.4% (Jordan) in EMR; 1.6% (Tajikistan) to 29.0% (Bulgaria) in EUR; 5.7% (Paraguay) to 23.7% (Argentina) in AMR; 3.4% (Indonesia) to 15.4% (Timor-Leste) in SEAR; and 3.0% (Mongolia) to 9.1% (Philippines) in WPR. Males had a higher prevalence of current tobacco smoking in 38 countries (p<0.05); females had a significantly higher prevalence of current tobacco smoking in one country (Portugal) (p<0.05).

Among the 51 countries in which the desire to quit was assessed among current tobacco smokers, the proportion of students who desired to quit ranged from 32.1% (Uruguay) to 90.2% (Philippines); the proportion of current tobacco smokers who reported a desire to quit exceeded 50% in 40 of those countries (Figure). By WHO region, the proportions ranged from 62.2% (Seychelles) to 86.3% (Kenya) in AFR; 49.1% (United Arab Emirates) to 75.8% (Yemen) in EMR; 43.5% (Italy) to 83.1% (Moldova) in EUR; 32.1% (Uruguay) to 70.1% (Guyana) in AMR; 67.8% (Timor-Leste) to 88.2% (Indonesia) in SEARO; and 66.9% (South Korea) to 90.2% (Philippines) in WPR.

**FIGURE:**
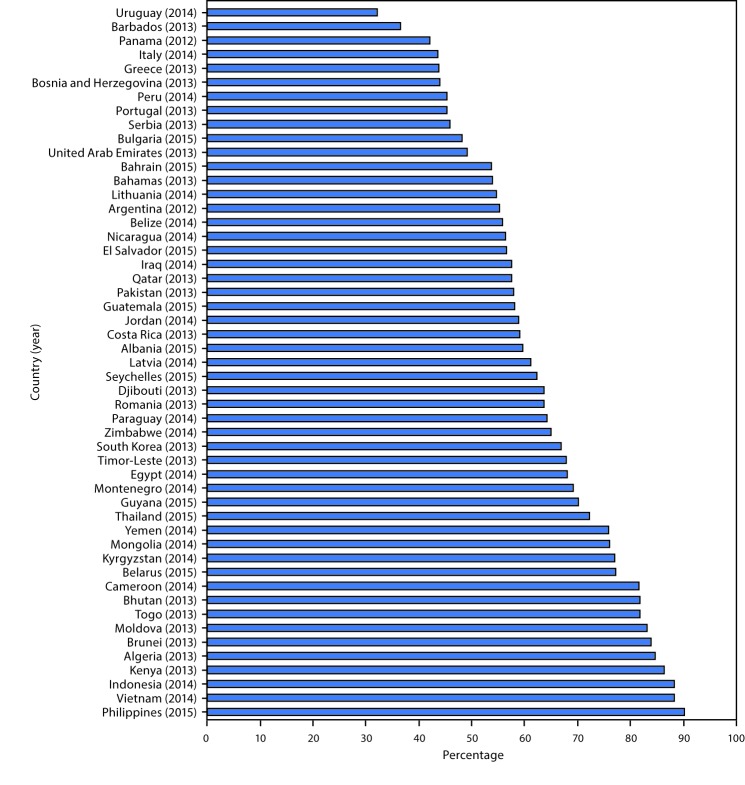
Proportion of current tobacco smokers* who desire to quit,^†^ among students aged 13–15 years — 51^§^ countries, Global Youth Tobacco Survey, 2012–2015 * Current tobacco smoking was defined as answering ≥1 day to the question “During the past 30 days, on how many days did you smoke cigarettes?” and/or “Yes” to “During the past 30 days, did you use any form of smoked tobacco products other than cigarettes (such as [country fills appropriate examples])?” ^†^ Desire to quit was defined as answering “Yes” to the question “Do you want to stop smoking now?” among current tobacco smokers. ^§^ Data not reported for desire to quit in Comoros (2015), Gabon (2014), Mozambique (2013), Senegal (2013), Sudan (2014), Georgia (2014), Kazakhstan (2014), San Marino (2014), Tajikistan (2014), and Sri Lanka (2015) because unweighted sample size <35 or relative standard error >0.3.

## Discussion

The prevalence of current tobacco smoking among students aged 13–15 years in 61 countries ranged from 1.7% (Sri Lanka) to 35.0% (Timor-Leste). In 38 countries, tobacco smoking prevalence was significantly higher among males than females. In 40 of 51 countries that collected data about the desire to quit, the proportion of students who reported current tobacco smoking and desired to quit exceeded 50%.

WHO’s Framework Convention on Tobacco Control (FCTC), the first international treaty negotiated under the auspices of WHO and developed in response to the global tobacco epidemic, includes evidence-based measures that have the potential to reduce youth tobacco use (*5*). These measures include increasing the price of tobacco (Article 6), bans on tobacco advertising, promotions, and sponsorship (Article 13), promoting tobacco cessation (Article 14), addressing illicit trade of tobacco products (Article 15), and prohibiting the sale of tobacco products to and by minors (Article 16). At the beginning of 2017, 59 of 61 countries in this report had ratified the FCTC. However, varying levels of tobacco control policy implementation and other country-specific factors might influence access to tobacco and tobacco smoking prevalence (*6*).

To assist with implementation of FCTC, countries can implement WHO’s MPOWER package (*7*). MPOWER is a set of evidence-based interventions intended to reduce tobacco use, including 1) monitoring tobacco use and prevention policies; 2) protecting persons from tobacco smoke; 3) offering help to quit tobacco use; 4) warning about the dangers of tobacco use; 5) enforcing bans on tobacco sponsorship, promotion, and advertising; and 6) raising taxes on tobacco. When implemented as part of a comprehensive approach, these strategies can help reduce youth tobacco use (*3*,*4*,*8*).

This report is subject to at least four limitations. First, data were self-reported by students, which might result in misreporting of smoking behavior. Second, the data presented represent only youths who are enrolled in school, which might limit generalizability to all youths in these countries. Third, low response rates in some countries might have resulted in nonresponse bias. Finally, only a limited number of countries were assessed from each WHO region; thus, the findings in this report are not necessarily generalizable to all countries in the respective WHO regions.

The prevalence of tobacco smoking is high among youths in many countries. However, many students who currently smoke report that they desire to quit. Implementing the evidence-based measures outlined in WHO’s MPOWER package can help reduce tobacco use among youths, as well as the estimated 1 billion tobacco-related deaths projected to occur during the 21st century if current trends persist (*1*).

**Summary**

**What is already known about this topic?**

Smoked tobacco products, such as cigarettes and cigars, are the most common form of tobacco consumed worldwide and most tobacco smokers begin smoking during adolescence.

**What is added by this report?**

Global Youth Tobacco Survey data from 61 countries from 2012 to 2015 revealed that the median current tobacco smoking prevalence among students aged 13–15 years was 10.7%. Tobacco smoking prevalence differed by gender and varied across countries. In the majority of countries, over 50% of youth tobacco smokers desired to quit.

**What are the implications for public health practice?**

Implementing the evidence-based measures outlined in the World Health Organization’s MPOWER package can help reduce tobacco use among youths, as well as the estimated 1 billion tobacco-related deaths projected to occur during the 21st century if current trends persist.

## Footnotes

* The Global Youth Tobacco Survey uses a two-stage sample design to select schools with a probability of selection proportional to enrollment size. The classes within selected schools are randomly selected and all students in selected classes are eligible to participate in the survey. More information is available from https://nccd.cdc.gov/GTSSDataSurveyResources/Ancillary/Documentation.aspx?SUID=1&DOCT=1.

† Two countries (Finland and Bolivia) collected data in 2012 and did not use the updated GYTS methodology, and were excluded; two countries, (Bangladesh and Turkmenistan), did not meet the minimum established threshold for reporting results of sample size <35 or relative standard error >0.3, and were excluded; and one country (Russian Federation), collected subnational data and was excluded.

§ Algeria, Cameroon, Comoros, Gabon, Kenya, Mozambique, Senegal, Seychelles, Togo, and Zimbabwe.

¶ Bahrain, Djibouti, Egypt, Iraq, Jordan, Pakistan, Qatar, Sudan, United Arab Emirates, and Yemen.

** Albania, Belarus, Bosnia and Herzegovina, Bulgaria, Georgia, Greece, Italy, Kazakhstan, Kyrgyzstan, Latvia, Lithuania, Moldova, Montenegro, Portugal, Romania, San Marino, Serbia, and Tajikistan.

†† Argentina, Bahamas, Barbados, Belize, Costa Rica, El Salvador, Guatemala, Guyana, Nicaragua, Panama, Paraguay, Peru, and Uruguay.

§§ Bhutan, Indonesia, Sri Lanka, Thailand, and Timor-Leste.

¶¶ Brunei, South Korea, Mongolia, Philippines, and Vietnam.

*** Past 30-day use of cigarettes was assessed with the following question, “During the past 30 days, on how many days did you smoke cigarettes?” and response option were: “a) 0 days,” “b) 1 or 2 days,” “c) 3 to 5 days,” “d) 6 to 9 days,” “e) 10 to 19 days,” “f) 20 to 29 days,” and “g) All 30 days.”

††† Past 30-day use of any form of smoked tobacco other than cigarettes was assessed with the following question, “During the past 30 days, did you use any form of smoked tobacco products other than cigarettes (such as [country fills approximate examples])?” and response options were: “a) Yes” and “b) No.”

§§§ Desire to quit smoking was assessed with the following question, “Do you want to stop smoking now?” and response options were: “a) I have never smoked,” “b) I don’t smoke now,” “c) Yes,” and “d) No.”
